# Laryngospasm During Pulsed Field Ablation of the Left Superior Pulmonary Vein

**DOI:** 10.1016/j.jaccas.2024.103095

**Published:** 2025-02-05

**Authors:** Bartosz Żuchowski, Małgorzata Połetek, Mateusz Dziarmaga, Adrianna Jęchorek, Jakub Szrama, Przemysław Guzik, Andrzej Minczykowski, Andrzej Wykrętowicz

**Affiliations:** a Department of Cardiology—Intensive Therapy, Poznan University of Medical Sciences, Poznań, Poland; b Department of Anesthesiology and Intensive Care, Poznan University of Medical Sciences, Poznań, Poland

**Keywords:** atrial fibrillation, anesthesia, complication, laryngospasm, pulmonary vein isolation, pulse field ablation

## Abstract

**Background:**

Pulsed field ablation (PFA) is an innovative, nonthermal, and effective method for pulmonary vein isolation, with a favorable safety profile in the treatment of atrial fibrillation (AF).

**Case Summary:**

A 74-year-old woman underwent elective pulmonary vein isolation with PFA for paroxysmal AF under general anesthesia with a laryngeal mask airway. During PFA delivery to the left superior pulmonary vein, a vocal cord spasm occurred, requiring immediate airway intervention. Ventilation was restored with a muscle relaxant followed by endotracheal intubation.

**Discussion:**

Although muscle contractions and vasospasms have been documented previously during PFA, this case is the first to be reported of laryngospasm. We hypothesize that it may result from direct stimulation of the recurrent laryngeal or vagus nerve owing to the superior vein course and deep catheter positioning.

**Take-Home Message:**

Because laryngospasm may occur, preparation for acute airway obstruction management is essential during PFA for AF.

## Graphical Abstract

**Figure:**
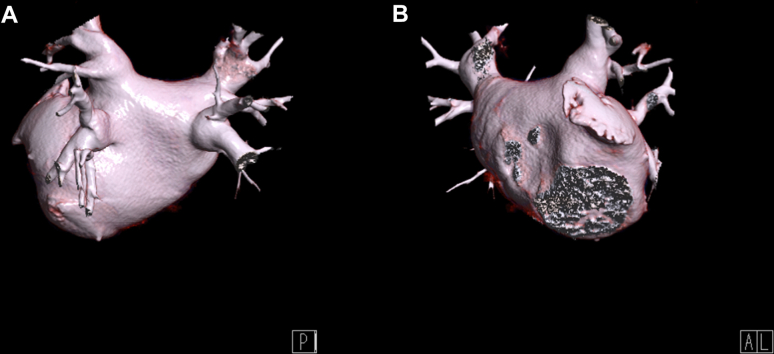

## History of Presentation

A 74-year-old woman was admitted to Poznan University Clinical Hospital for an elective pulmonary vein isolation (PVI) procedure owing to paroxysmal atrial fibrillation. She had been diagnosed with atrial fibrillation 10 years earlier, with a recent, significant increase in the frequency of arrhythmia episodes.

**Take-Home Messages**

• Be aware that PFA can induce airway spasm, which may necessitate the urgent administration of muscle relaxants and endotracheal intubation.
• During PFA ablation, extra care should be taken when targeting PVs with ostia located high in the chest, preferably avoiding deep catheter positioning owing to the possible proximity to the RLN.

On examination, the patient exhibited sinus bradycardia at 55 beats/min. Blood pressure and laboratory results were within normal ranges. She had a rather slim physique, with a body mass index of 18.8 kg/m^2^.

**Figure 1:**
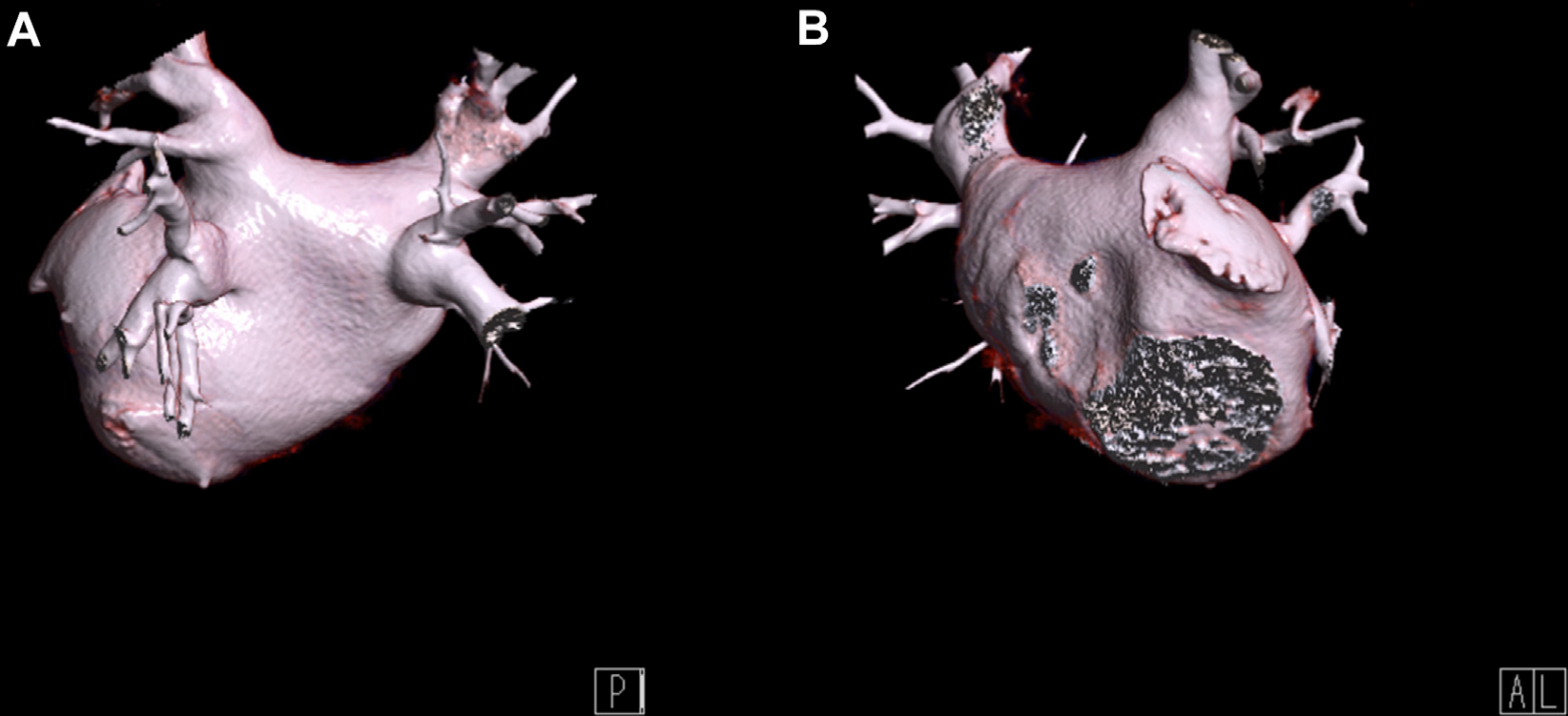
Computed Tomography Scan of the Left Atrium and Pulmonary Veins The figure illustrates the anatomy of the left atrium and pulmonary veins as obtained from computed tomography before the procedure. (A) Posterior view. (B) Left anterior view.

Cardiac echocardiography showed mild dilatation of the left atrium (27 cm^2^) and normal right atrium dimension (16 cm^2^). The left ventricle ejection fraction was normal at 67%. A computed tomography scan performed before ablation (Figure 1) ruled out the presence of a left atrium thrombus and enabled measurement of the pulmonary vein (PV) ostia diameters (Table 1).

**Table 1:** Computed Tomography Scan Dimensions

Anatomical Structure	Dimension (Width × Height), mm
LSPV	21 × 21
LIPV	19 × 9
RSPV	23 × 20
RIPV	19 × 17
Left atrium	91 × 45

LIPV = left inferior pulmonary vein; LSPV = left superior pulmonary vein; RIPV = right inferior pulmonary vein; RSPV = right superior pulmonary vein.

## Past Medical History

The patient’s medical history included Hashimoto’s disease in a state of euthyreosis, hypertension, sciatica, and depression. Her regular medications included levothyroxine 75 μg/day, bisoprolol 7.5 mg/day, escitalopram 5 mg/day, trazodone 75 mg/day, and apixaban 10 mg/day.

## Investigations

**Figure 2:**
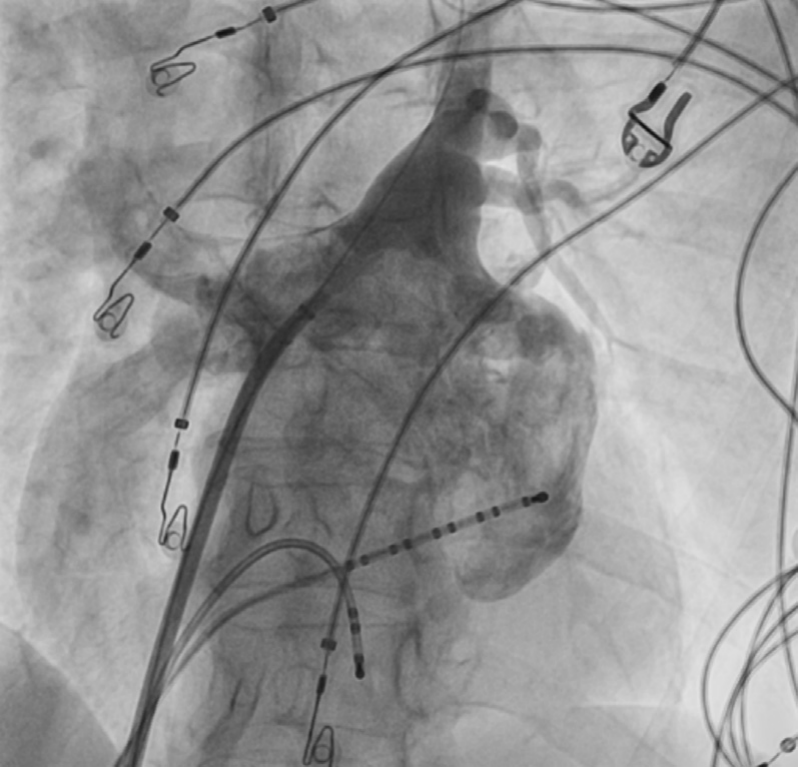
Left Atrium Angiography Angiography of the left atrium performed during the procedure. Left anterior oblique 30° view.

**Figure 3:**
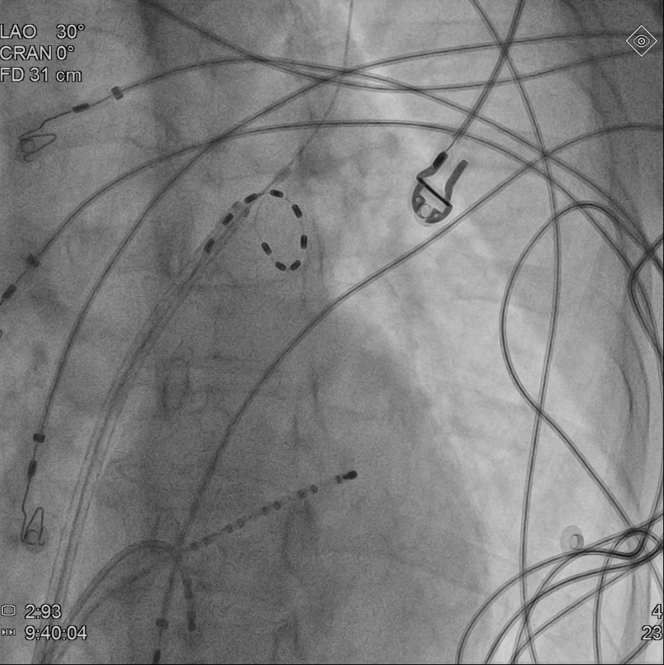
Deployment of the PulseSelect Catheter The PulseSelect catheter is deployed in the region of the left superior pulmonary vein. Left anterior oblique 30° view.

**Figure 4:**
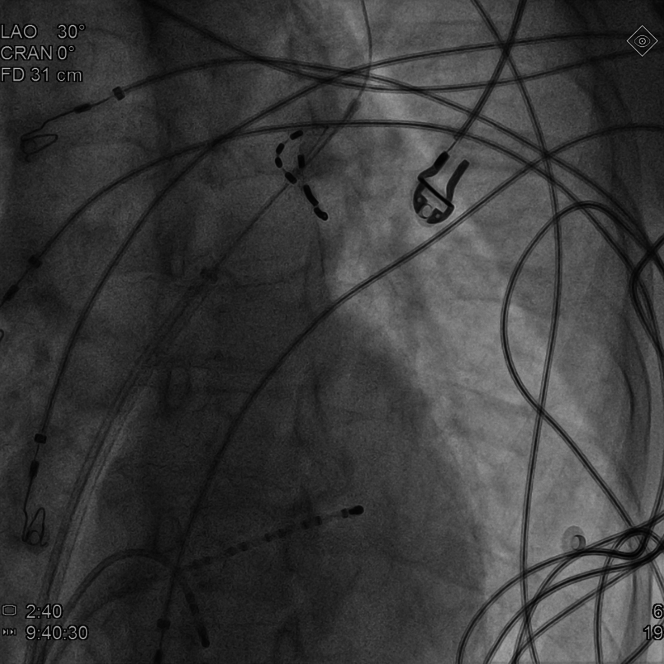
Position of the PulseSelect Catheter During Application Position of the PulseSelect catheter during the application of pulsed field ablation in the left superior pulmonary vein. This position resulted in the occurrence of laryngospasm. Left anterior oblique 30° view.

**Figure 5:**
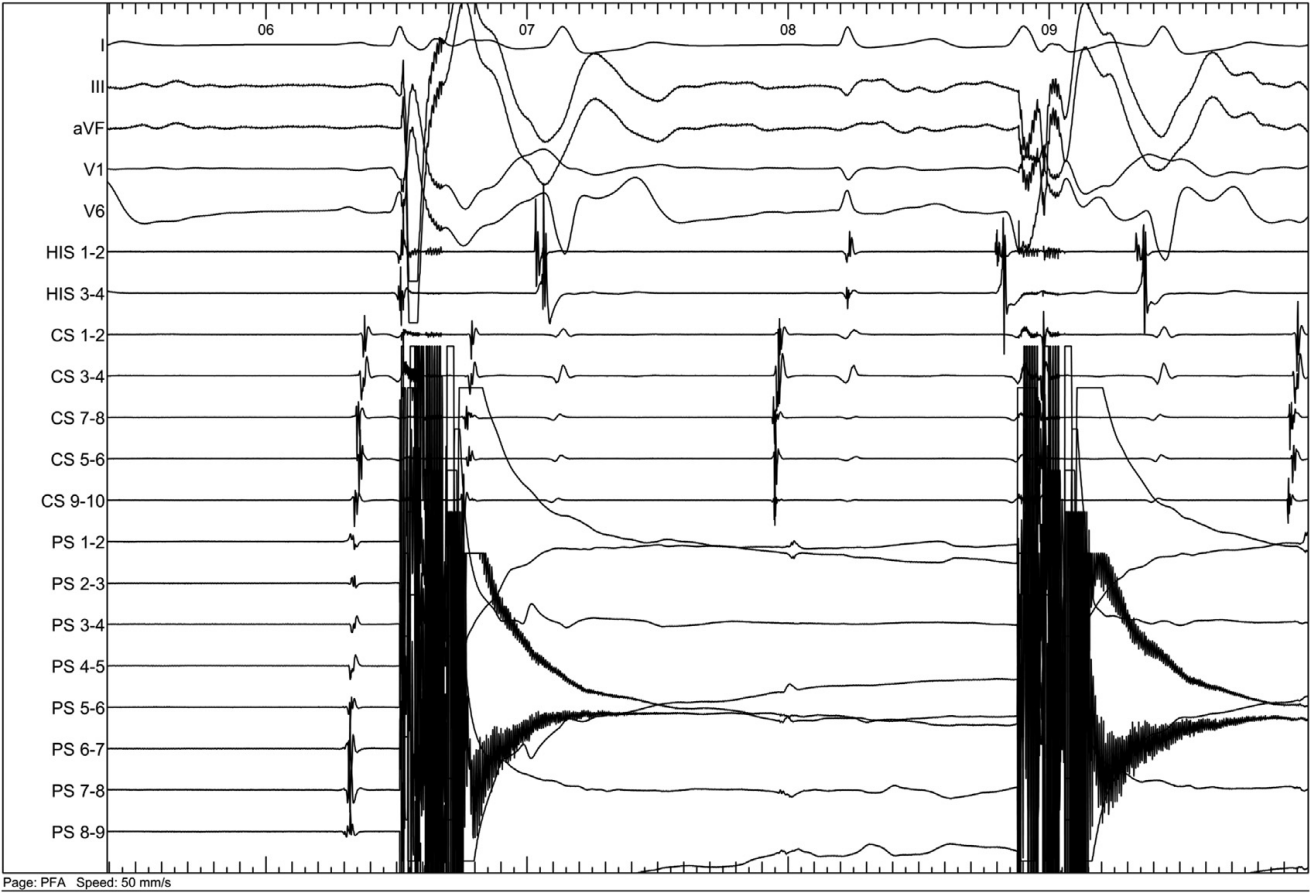
Pulse Field Ablation in the Left Superior Pulmonary Vein The first application resulted in immediate disappearance of pulmonary vein potentials, visible on every PulseSelect catheter electrode.

The patient was scheduled for PVI using the PulseSelect (Medtronic) pulsed field ablation (PFA) system. This novel 9-F, 25-mm loop catheter features 9 electrodes that enable sensing, pacing, and delivery of bipolar, biphasic, high-power (1,500-V) electrical pulses, creating lesions around the PVs through irreversible electroporation. The PVI protocol with PulseSelect catheter involves 4 ostial and 4 antral applications per vein.^1^ Owing to pain and muscle contractions during PFA, the procedure was performed under general anesthesia, supervised by an anesthesiologist. Mechanical ventilation was maintained with a laryngeal mask airway (LMA). Through femoral vein punctures, a transseptal sheath (SL0 Swartz, Abbott) and 2 diagnostic catheters—a decapolar to the coronary sinus (Dynamic XT, Boston Scientific) and quadripolar to the right ventricle (Viking, Boston Scientific)—were introduced. The transseptal puncture was performed under fluoroscopic guidance with a BRK-1 needle (Abbott). A nonselective angiogram of the left atrium and PVs was obtained by injecting 15 mL of iodine contrast during rapid ventricular pacing (Figure 2, Video 1). The left superior PV (LSPV) was found to be directed upwards, with a high ostium location. After exchanging the transseptal sheath for a 10-F steerable FlexCath Contour, the PulseSelect catheter was deployed to the LSPV ostium (Figure 3, Video 2). The first PFA application was delivered with the middle (fifth) electrode at the 6 o'clock position (Figure 4, Video 3). Disappearance of sharp atrial electrograms was observed on each electrode pair (Figure 5), and left diaphragm contractions were also noted (Video 4). Immediately after the first PFA application, the ventilation became ineffective.

## Differential Diagnosis

Ineffective ventilation was indicated by a sudden drop in end-tidal carbon dioxide concentration, increased peak inspiratory pressure, and failure to deliver tidal volume. After confirming the correct LMA position, the anesthesiologist attempted manual ventilation with an anesthesia bag. Because ventilation remained ineffective, the LMA was removed for a laryngoscopic inspection, revealing a complete vocal cord spasm obstructing the larynx. A drop in peripheral oxygen saturation to 70% was observed. Meanwhile, the patient's blood pressure was confirmed to be normal, and a quick echocardiogram revealed no abnormalities. On fluoroscopy, the ablation catheter's guidewire was not trapped and moved freely in the LSPV.

## Management

To relieve the laryngospasm, 80 mg of suxamethonium chloride (Chlorsuccillin, Bausch Health) was administered intravenously. After successful relaxation of the vocal cords, bag-valve-mask ventilation effectively provided oxygenation. Subsequently, intubation with an endotracheal tube was performed to secure the airway and prevent recurrent laryngospasm. The PVI with PFA then proceeded without complications. No further ventilation issues were observed. Diaphragmatic contractions were noted during every application in both the left and right superior veins. Both entry and exit blocks were confirmed in sinus rhythm after a 15-minute wait. Emergence from anesthesia was smooth, and the patient was transferred to the cardiology department in good condition.

## Outcome and Follow-Up

The day after the procedure, the patient complained only of a sore throat. She was breathing normally, without stridor or dyspnea, and reported no chest or abdominal pain, only mild discomfort at the puncture site. Fiberoptic laryngoscopy revealed swelling, redness, and a minor laceration on the right arytenoid, which are common after endotracheal intubation. The larynx was unchanged anatomically and functionally; the vocal folds were smooth, properly and symmetrically mobile; and the airway was wide. Laboratory results showed no signs of significant hemolysis or renal failure. Troponin I concentration was significantly elevated (2,809.4 ng/L; reference value, <15.60 ng/L), as expected after PVI. The patient remained in sinus rhythm until discharge.

## Discussion

PFA is an innovative, nonthermal cardiac ablation method that improves the safety of PVI. Unlike thermal ablation methods, PFA uses electric field pulses that preferentially target cardiomyocytes, reducing the risk of collateral damage to surrounding structures, which can lead to complications such as phrenic nerve paresis, atrioesophageal fistula, or PV stenosis.^1^, ^2^, ^3^ PFA has demonstrated comparable efficacy to thermal ablation in achieving durable PVI, with an encouraging safety profile.^4^^,^^5^ However, adopting new technologies inherently requires addressing specific procedural challenges and learning associated intricacies.

PFA uses high-energy pulses to create lesions in cardiac tissue by forming nanopores in cell membranes, ultimately inducing its necrosis or apoptosis. It spares the stromal cells and preserves the extracellular matrix. One of the rare complications reported in association with PFA is coronary artery spasm.^5^^,^^6^ The mechanism behind this phenomenon is not well understood, though available data suggest a direct correlation with the proximity of the PFA catheter to coronary vessels. Coronary artery spasms have been reported primarily when a PFA catheter was used in the cavotricuspid isthmus or mitral isthmus. Additionally, PFA can induce muscle contractions, necessitating deep sedation or general anesthesia during the procedure. These contractions are believed to result from direct stimulation of muscles or nerves.^7^

Laryngospasm is a potentially life-threatening condition characterized by the sustained closure of the vocal cords, resulting in airway obstruction.^8^ This reflex serves as a protective mechanism against pulmonary aspiration. The motor response involves the contraction of intrinsic laryngeal muscles: the lateral cricoarytenoid, thyroarytenoid, and posterior cricoarytenoid muscles, all of which are innervated by the inferior laryngeal nerve—a branch of the recurrent laryngeal nerve (RLN).^8^ The risk of laryngospasm may increase owing to anesthesia, patient-specific, and procedure-related factors. For example, the risk is higher when a LMA, facemask, or volatile anesthetics such as desflurane are used. Regarding patients, children, asthmatics, smokers, and individuals with sleep apnea or gastroesophageal reflux are at increased risk. Surgeries involving tissues near the larynx or related distal afferent nerves, such as esophageal, bronchial, hypospadias correction, appendectomy, or cervical dilation procedures are more likely to trigger laryngospasm. Psychogenic laryngospasm has also been reported.

In the present case, laryngospasm resulted in significant desaturation owing to ineffective ventilation. A prompt response from the anesthesiologist restored the airway. In some centers, PFA is performed under deep sedation without the presence of an anesthesiologist. In such situations, when laryngospasm occurs, the person responsible for sedation should consider calling an emergency team immediately. Urgent management of laryngospasm may require oxygen supplementation and positive pressure ventilation, deepening sedation, administering muscle relaxants, or even performing a rescue tracheostomy if the previous methods do not relieve the spasm.

**Figure 6:**
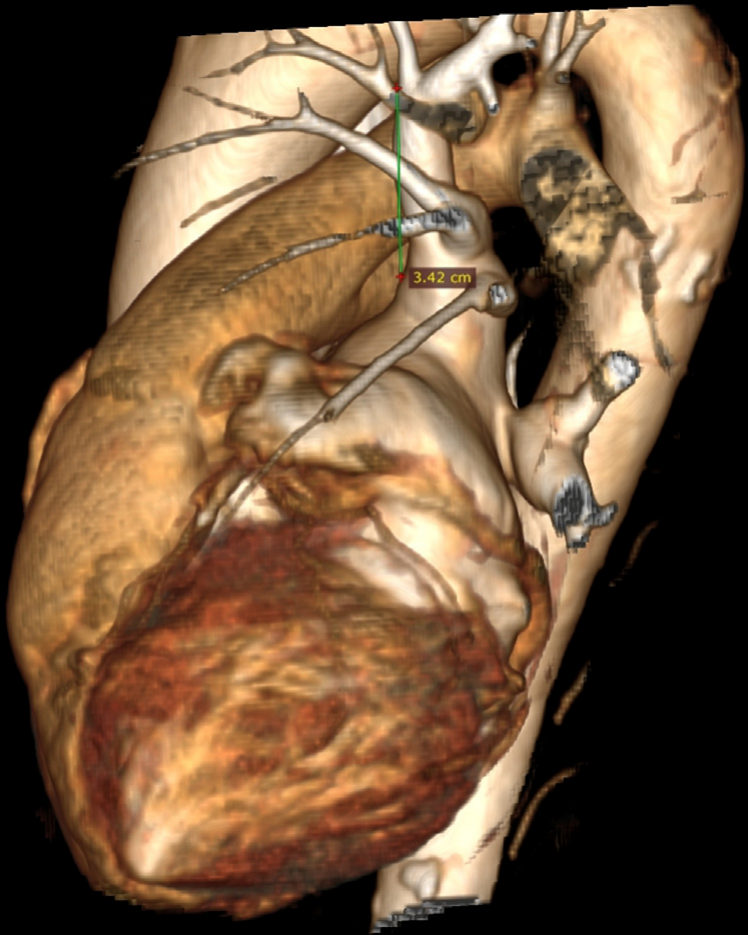
Computed Angiography The computed angiography reveals the proximity of the left superior pulmonary vein ostium to the aortic arch. Lateral view.

**Figure 7:**
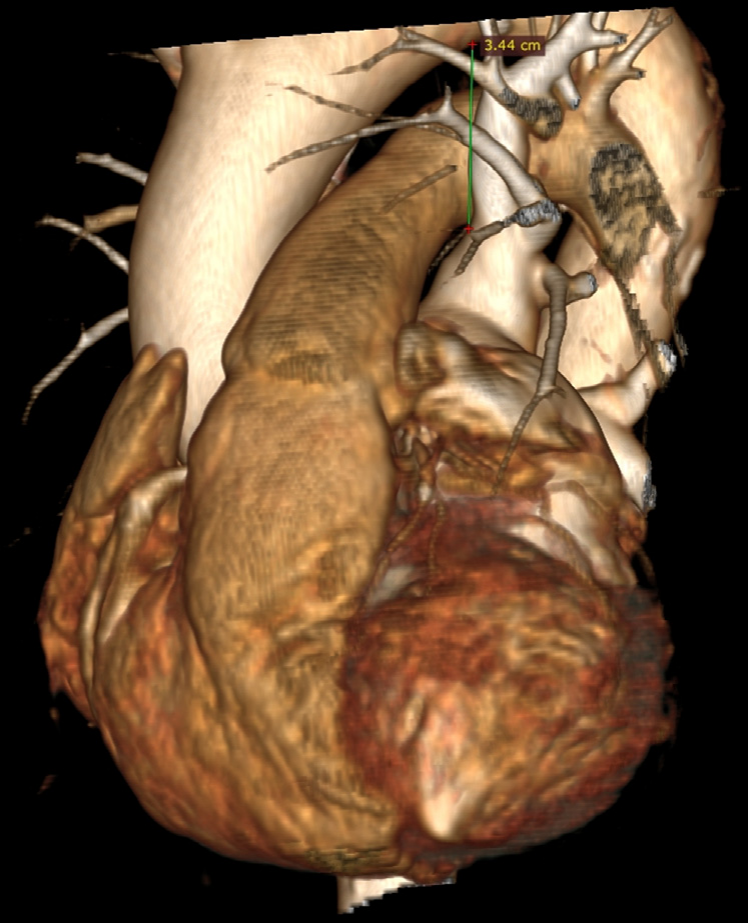
Computed Angiography Computed angiography reveals the proximity of the left superior pulmonary vein ostium to the aortic arch. Anterolateral view.

**Figure 8:**
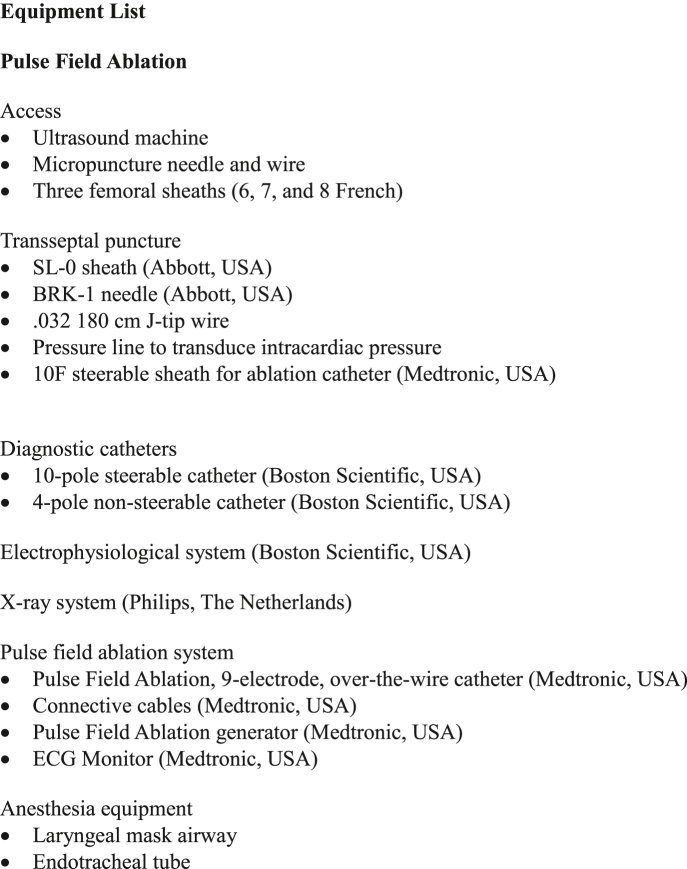
Equipment List Sample equipment list to perform pulse field ablation of pulmonary veins.

To our knowledge, there have been no previous reports of laryngospasm occurring during PFA. However, transient vocal cord paralysis owing to RLN injury has been described previously as a rare complication of radiofrequency ablation.^9^ We hypothesize that, in our patient, the likely mechanism was direct stimulation of the left RLN during PFA application in the LSPV. The left RLN branches off from the vagus nerve at the level of aortic arch, passing anteriorly, wrapping underneath, and then continuing posteriorly. Given that the LSPV ostium was located high, the catheter was positioned deep, at the level of the left bronchus, and the patient was relatively slim, it is plausible that the high-energy pulses affected the RLN or its branches, leading to laryngeal muscle response. Although the computed tomography scan does not allow for direct nerve visualization, the distance between the lowest point of the aortic arch and the LSPV ostium, was <3.5 cm (Figures 6 and 7). The distance from the catheter to the RLN was likely even shorter. The patient may have had an anatomical variation of the RLN course that brought it closer to the LSPV. Other nerve structures potentially involved include distal branches of the vagus nerve contributing to the cardiac plexus. The exact nerve and muscle stimulation range for the PulseSelect catheter is not well-established. Previous studies suggest that the stimulation range depends on the electrical impulse's power, duration, and polarity (Figure 8).^10^

## Conclusions

This report of laryngospasm during PFA in the LSPV is the first. Medical personnel performing ablations with PFA should be aware of this potentially life-threatening complication. Physicians responsible for maintaining the sedation or anesthesia during PFA procedures must be adequately prepared and trained to urgently administer muscle relaxants and perform endotracheal intubation with endotracheal tube to prevent hypoxia.

## Funding Support and Author Disclosures

Dr Żuchowski received consulting and lecture fees from Medtronic, the manufacturer of the PulseSelect catheter. All other authors have reported that they have no relationships relevant to the contents of this paper to disclose.

## Appendix

Video 1 Left Atrium Angiography Angiography of the left atrium performed during the procedure. Left anterior oblique 30° view.

Video 2 Deployment of the PulseSelect Catheter The PulseSelect catheter is deployed in the region of the left superior pulmonary vein. Left anterior oblique 30° view.

Video 3 Positioning of the PulseSelect Catheter Before Application Advancement of the PulseSelect catheter to the left superior pulmonary vein to perform pulse field ablation. This position resulted in the occurrence of laryngospasm. Left anterior oblique 30° view.

Video 4 Diaphragm Contractions During Delivery of Pulse Field Ablation Contractions of diaphragm during pulse field ablation of the left superior pulmonary vein.
